# The Promigratory Activity of the Matricellular Protein Galectin-3 Depends on the Activation of PI-3 Kinase

**DOI:** 10.1371/journal.pone.0029313

**Published:** 2011-12-28

**Authors:** Fabiana H. M. Melo, Diego Butera, Mara de Souza Junqueira, Daniel K. Hsu, Ana Maria Moura da Silva, Fu-Tong Liu, Marinilice F. Santos, Roger Chammas

**Affiliations:** 1 Departamento de Radiologia e Oncologia, Faculdade de Medicina da Universidade de São Paulo, São Paulo, São Paulo, Brazil; 2 Laboratório de Imunopatologia, Instituto Butantan, São Paulo, São Paulo, Brazil; 3 Department of Dermatology, University of California Davis, Davis, California, United States of America; 4 Departamento de Biologia Celular e do Desenvolvimento, Instituto de Ciências Biomédicas, Universidade de São Paulo, São Paulo, São Paulo, Brazil; 5 Instituto do Cancer do Estado de São Paulo, São Paulo, São Paulo, Brazil; Chinese University of Hong Kong, Hong Kong

## Abstract

Expression of galectin-3 is associated with sarcoma progression, invasion and metastasis. Here we determined the role of extracellular galectin-3 on migration of sarcoma cells on laminin-111. Cell lines from methylcholanthrene-induced sarcomas from both wild type and galectin-3^−/−^ mice were established. Despite the presence of similar levels of laminin-binding integrins on the cell surface, galectin-3^−/−^ sarcoma cells were more adherent and less migratory than galectin-3^+/+^ sarcoma cells on laminin-111. When galectin-3 was transiently expressed in galectin-3^−/−^ sarcoma cells, it inhibited cell adhesion and stimulated the migratory response to laminin in a carbohydrate-dependent manner. Extracellular galectin-3 led to the recruitment of SHP-2 phosphatase to focal adhesion plaques, followed by a decrease in the amount of phosphorylated FAK and phospho-paxillin in the lamellipodia of migrating cells. The promigratory activity of extracellular galectin-3 was inhibitable by wortmannin, implicating the activation of a PI-3 kinase dependent pathway in the galectin-3 triggered disruption of adhesion plaques, leading to sarcoma cell migration on laminin-111.

## Introduction

Activation of the migratory and invasive capacities by tumor cells are often associated with tumor progression [1]. Central to these changes are alterations in either the expression pattern or activity of integrins, key regulators of the organization of cytoskeleton, cell adhesion and survival [2]. RAS pathway activation promotes invasiveness and metastatic spread in different cancers, including sarcomas. We have previously shown that EJ-*ras* expressing NIH-3T3 fibroblasts acquired the ability to migrate on laminin-111 surfaces [3], [4]. Although fibroblasts are highly migratory cells, this is usually true for the migration elicited by interstitial extracellular matrices composed mainly of collagen I and III and fibronectin. Migration on laminin is considered a dysfunctional pattern of migration for fibroblasts [5]. We showed that this pattern was dependent on the expression of α6β1 integrin, whose expression is up-regulated after transfection with activated *ras*. Not only the expression of the integrins is altered upon transformation, but their pattern of glycosylation also varies [6]. Integrins in transformed cells tend to accumulate tri- and tetraantennary *N*-linked oligosaccharides recognized by the plant lectin leucoagglutinin from *Phaseolus vulgaris* (L-PHA) [7]–[9]. These *N*-linked glycans may present polylactosamine structures, which are among the glycans recognized by the animal lectin, galectin-3 [10]–[12].

Galectin-3 is the only chimeric member of the family of galectins [13]. It is a 29–35 kDa protein which has a unique amino-terminal domain involved in the translocation of galectin-3 within different cellular compartments [14], a proline, glycine and tyrosine rich region and a carbohydrate recognition domain (CRD) in the carboxy-terminal region. Galectin-3 is mainly a cytoplasmic protein that can translocate into the nucleus, mitochondria and it can even be secreted [15]–[17]. Ochieng and colleagues demonstrated that while detached and spherical cells secrete galectin-3 constitutively, attached and spread cells take galectin-3 up from the conditioned medium [18]. These results support the notion that mechanotransductional signals may be involved in galectin-3 secretion. In the extracellular space, galectin-3 binds to a variety of extracellular matrix components like laminin, fibronectin, hensin, elastin, collagen IV and tenascin-C and -R [19], modulating cell-matrix dependent biological processes, such as adhesion and migration, in normal and cancer cells. In this context, galectin-3 has all features of a matricellular protein [20].

In cancers, galectin-3 expression is dependent on the tumor histogenesis [20], [21]. An increase in galectin-3 expression has been reported in lymphomas, head and neck and thyroid carcinomas, glioblastomas and osteosarcomas [20], [22]–[25]. In contrast, down-regulation of galectin-3 expression has been reported in breast and prostate cancer [26], [27]. Changes in galectin-3 expression have also been observed in colon cancer [28], [29]. Accumulation of galectin-3 in transformed highly migratory fibroblasts and invasive osteosarcomas [4], [22], [28], [29] prompted us to investigate its possible role as a modulator of integrin function in tumor cells of mesenchymal origin.

Here we have addressed the role of galectin-3 in the adhesive and migratory properties of methylcholanthrene-induced sarcoma-derived cells from both galectin-3^+/+^ and galectin-3^−/−^ mice on laminin-111 substrata. We showed that galectin-3 decreased cell adhesion and promoted cell migration. Furthermore, addition of extracellular galectin-3 to galectin-3^−/−^ cells induced recruitment of SHP-2 phosphatase to and loss of phosphorylated FAK and phospho-paxillin from focal complexes. The promigratory activity of extracellular galectin-3 was associated with AKT phosphorylation, and it was PI3-kinase dependent, since it could be inhibited by wortmannin.

## Results

### Cytoplasmic galectin-3 is recruited to the leading edge of migrating cells

**Figure 1:**
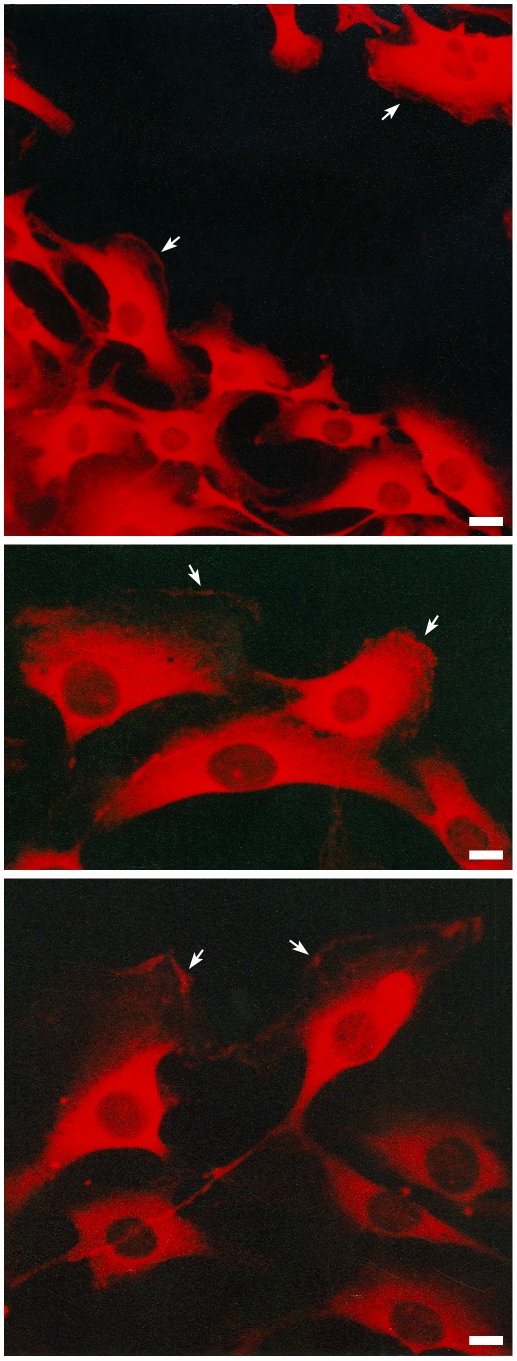
Galectin-3 is found on the lamellipodia of migrating fibroblasts. CCR2 cells were subjected to the scrape assay. The cells were grown in coverslips to confluence, when a wound was made. The cells were maintained in culture for additional 24 hours, when it was possible to observe cells migrating into the wounded area. Panels show the distribution of galectin-3, which was detected with galectin-3 monoclonal antibody M3/38, followed by incubation with a rhodamine-conjugated secondary antibody. Galectin-3 is localized in the cytoplasm of the cells and also in leading edge of migrating cells as indicated by arrows.

In a previous report [4], we have shown that EJ-ras expressing NIH3T3 cells (CCR2 cells) acquired the ability to migrate on laminin-111 surfaces, characterizing a dysfunctional pattern of migration. Migration on laminin-111 is dependent on α6β1 integrins, whose expression is up-regulated in CCR2 cells. Subcellular localization of galectin-3 in migrating CCR2 cells was analyzed as shown in Fig. 1, using M3/38 monoclonal antibody, whose epitope maps within residues 48–100 in the amino-terminal domain of galectin-3 [30]. Migrating cells displayed galectin-3 in the lamellipodia, as indicated by arrows in the illustrative Fig. 1. We have also previously shown that experimental sarcomas expressed galectin-3, galectin-3 ligands and acquired the capacity of migrating onto basement membranes of muscle fibers, a commonly observed feature of locally invasive sarcomas [12]. Altogether these results suggest the involvement of galectin-3 in the acquisition of the dysfunctional migration pattern of sarcoma cells on basement membrane proteins, such as laminin.

### Establishment and characterization of sarcoma-derived cell lines from both wild type and galectin-3^−/−^ mice

**Figure 2:**
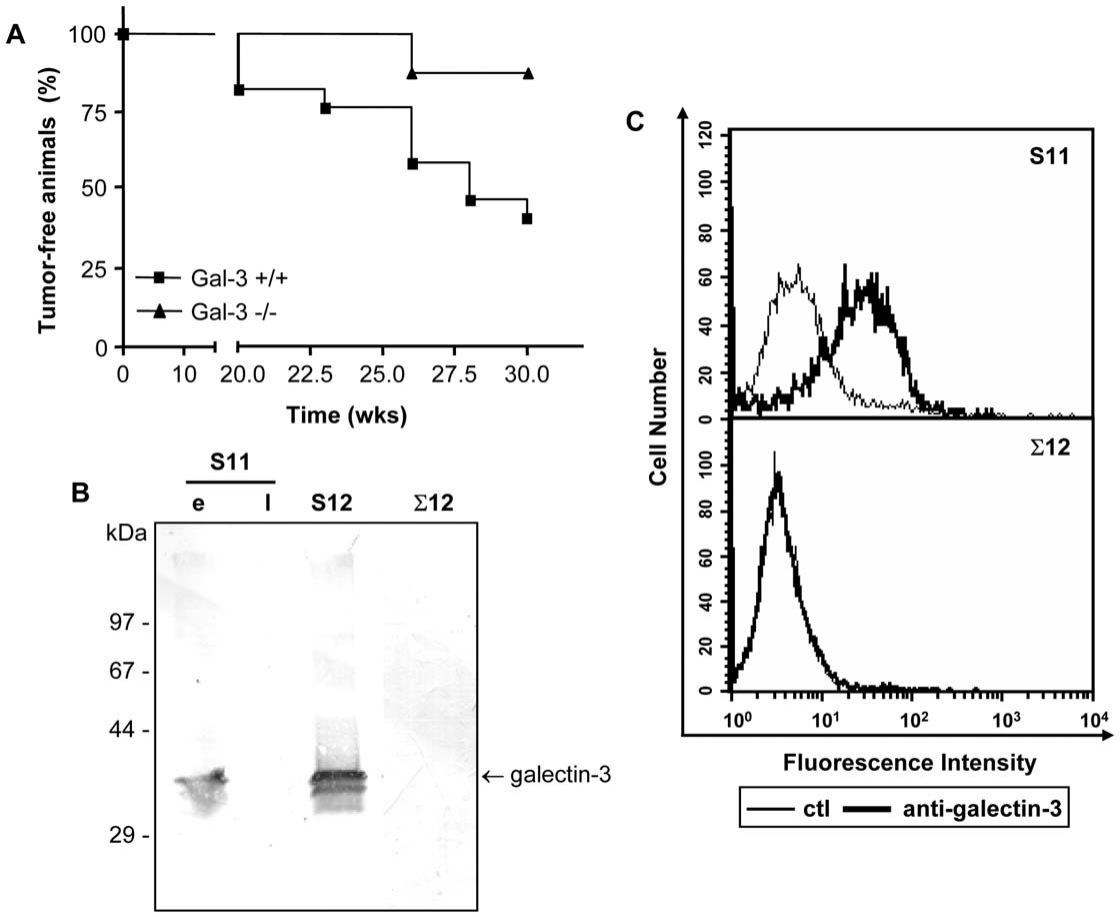
Galectin-3^−/−^ mice are more resistant to methylcholanthrene induced sarcomas development. Wild type and galectin-3^−/−^ mice were given two subcutaneous doses of 3-methylcholanthrene, a known chemical carcinogen. The development of tumors was followed weekly after the second dose. Analysis of the disease-free survival curves from both wild type (n = 23 animals) and galectin-3^−/−^ mice (n = 17 animals) showed that wild type mice developed sarcomas faster than galectin-3^−/−^ animals (**A**) (*Log-rank* test, p = 0.04). At necropsy, tumors were maintained in culture conditions, allowing for the establishment of sarcoma derived cell lines. Galectin-3 accumulation was analyzed in protein extracts from established cell lines from both wild type (S11 and S12) and galectin-3^−/−^ mice (Σ12) by western blotting. S11 and S12 cells, but not Σ12 cells, expressed galectin-3. While S12 cells maintained high expression of galectin-3 in all passages analyzed, only early (e, passage number<15), but not late (l, passage number>15) passages of S11 expressed galectin-3. Note that part of the molecules produced were further processed rendering the lower molecular weight form of the lectin, which appears as a 30 kDa band (**B**). The expression of galectin-3 on S11 and Σ12 cell surface was analyzed by flow cytometry. S11 cells display at least part of its galectin-3 content on the cell surface (**C**).

In order to investigate the role of galectin-3 in dysfunctional migration of sarcoma cells, sarcoma-derived cell lines from both wild type and galectin-3^−/−^ mice were established. Sarcomas were chemically induced by subcutaneous injections of methylcholanthrene [31]. As shown in Fig.2A, treatment of either wild type C57Bl/6 mice (n = 23) or galectin-3^−/−^ mice (n = 17) with two subcutaneous injections of methylcholanthrene (50 µg/animal/dose within a 12-week interval) led to the generation of palpable tumors within 7 months. A significant difference in the number of tumor-bearing animals after 7 months was observed. Galectin-3^−/−^mice tended to be more resistant to the development of tumors. Histopathological analysis showed that all tumors were sarcomas (Fig. S1). Sarcomas from both wild type and galectin-3^−/−^ mice were cultured and three independent cell lines were obtained, S11 and S12 (derived from sarcomas of wild type mice) and Σ12 (derived from a galectin-3^−/−^ sarcoma). Galectin-3 was found only in extracts of earlier passages of S11 cells. On the other hand, S12 cells maintained the expression of galectin-3 regardless of their passage number (Fig. 2B). The two bands present in S12 cell extracts are often found in these and other cellular extracts. A possible explanation for the lower apparent molecular weight band is cleavage of the amino-terminus region of galectin-3. This apparently cleaved form is also found in recombinant galectin-3 (see reference [30], for an example). The precise nature of the protein modification which yields the second band is currently under investigation in our laboratory. As expected, Σ12 cells did not express galectin-3 (Fig. 2B). Galectin-3 was found on the membrane of S11 cells (Fig. 2C). Similar results were observed for S12 cells (data not shown). All three clones displayed similar levels of both α6 and β1 integrin chains on the cell surface (Fig. S2). The α6β1 integrin was functional and mediated sarcoma cell adhesion and migration on laminin-111 (data not shown).

### Galectin-3 modified the adhesion and migration pattern of sarcoma-derived cell lines on laminin-111

**Figure 3:**
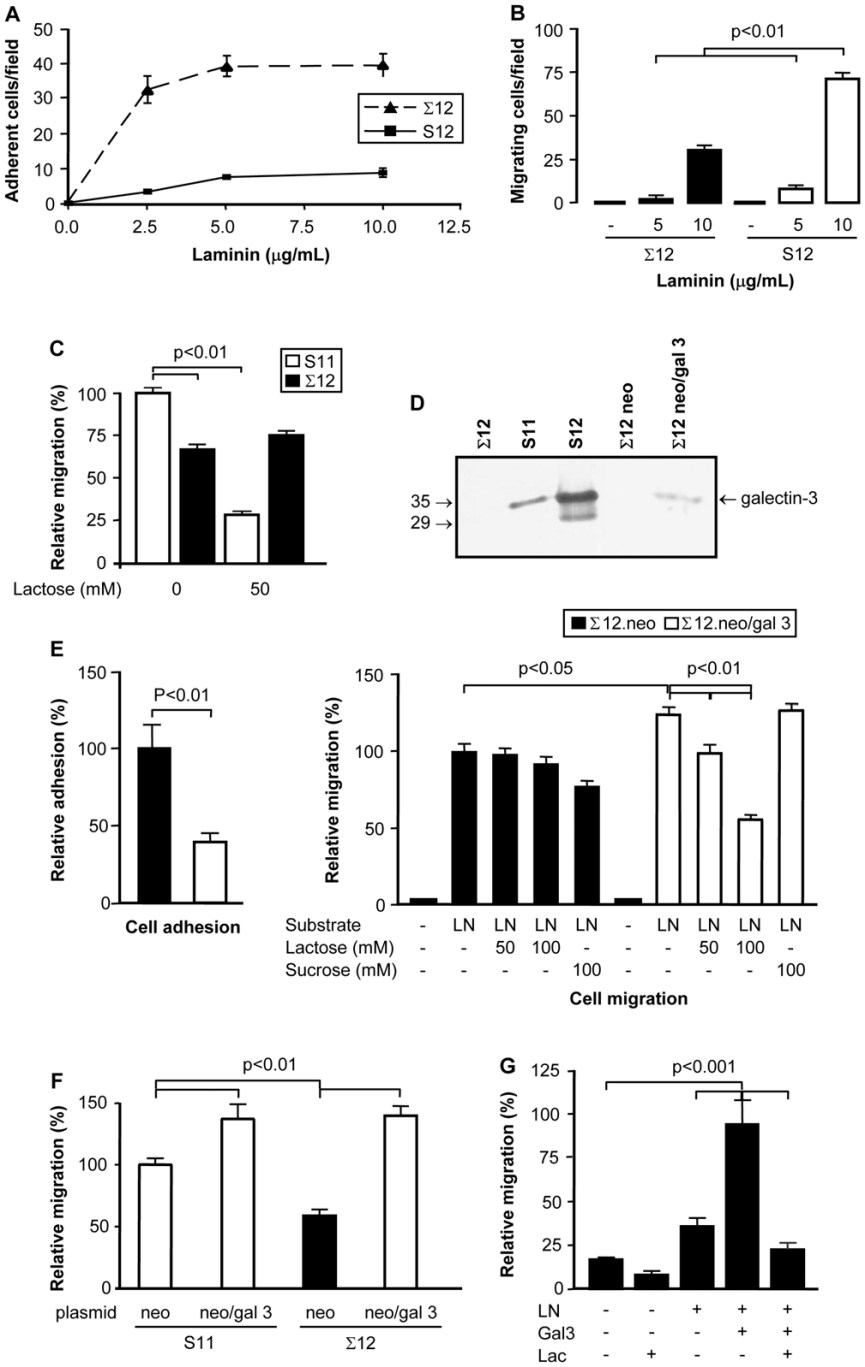
The galectin-3^−/−^ cell line is more adherent and less migratory on laminin-111 surfaces. Σ12 cells were compared to S12 cells regarding their adhesive (**panel A**) and migratory capacity (**panel B**). For the adhesion assay (**panel A**), each point represents the mean of triplicates, SD is also represented. The migratory capacity of S12 and Σ12 cells on laminin-111 was evaluated using Transwell chambers (**panel B**). Migration assays were also performed comparing S11 and Σ12 cells, as described in B. In these assays, lactose was used to inhibit cell migration. S11 cell migration, but not Σ12 cell migration was inhibited by lactose. Migration of S11 cells without lactose inhibition was used as internal reference for determining relative migration (**panel C**). Σ12 cells were transiently transfected with either a plasmid containing the galectin-3 gene or a control plasmid. While no galectin-3 was found in Σ12 cells transfected with the control plasmid (neo) expression of galectin-3 was observed in neo/gal-3 transfected cells by western blotting. Extracts of S11 and S12 cells used were used as positive controls (**panel D**)**.** Σ12 cells were rendered less adherent and more migratory upon transfection with pEF1-neo/gal-3. Relative adhesion was determined based on the adhesion of neo-transfected cells. Cell migration was evaluated in Transwell chambers, whose filters were coated with laminin-111. The transfected Σ12 cells were incubated in serum-free medium either in the absence or presence of the indicated concentrations of lactose or sucrose. Migration of the former cells towards laminin-111 was inhibited by lactose, but not by sucrose, indicating a role for galectin-3 in the modulation of cell migration in response to laminin-111 (**panel E**). S11 cells were transiently transfected with pEF1-neo or pEF1-neo/gal-3 and checked for their migratory response towards laminin-111 in Transwell chambers. A significant increase in the migratory response elicited by laminin-111 was observed in S11 cells overexpressing galectin-3. Migration of neo-transfected S11 cells was used as internal reference for determining relative migration (**panel F**). The migratory capacity of Σ12 cells was evaluated in absence or presence of extracellular galectin-3 (gal-3), laminin-111 (LN) and in the presence or absence of 100 mM lactose (Lac) for 24 hours using the scrape assay. Exogenous galectin-3 increased the migration of Σ12 cells when soluble LN was added, such increase was inhibited by lactose. Migration of Σ12 cells in the presence of both LN and galectin-3 was used as internal reference for determining relative migration (**panel G**). In all panels, results are representative of at least three independent assays. Means and SD are represented. White bars represent results from galectin-3 expressing cells; black bars represent results from galectin-3 null cells.

Both S11 and S12 cells adhered less on laminin-111 surfaces than Σ12 cells (Fig. 3A and data not shown). On the other hand, Σ12 were less migratory than S11 and S12 cells on laminin-111 substrata (Fig. 3B and 3C), indicating that galectin-3 modulated, at least in part, the interaction of sarcoma cells with laminin-111. Lactose inhibited significantly the laminin-induced migration of S11 cells (Fig. 3C).

Σ12 cells were then transiently transfected with galectin-3 in order to determine whether the migratory phenotype of galectin-3 expressing sarcoma cells could be restored. No changes in β1 integrin expression were observed in the transfected cells (neo, control; neo/gal 3, galectin-3 expressing cells) (Fig. S3). Galectin-3 expression in Σ12 cells transfected with neo/gal 3 was demonstrated by western blotting (Fig. 3D). The expression level of galectin-3 was estimated as a tenth of the amount expressed by S12 cells, thus indicating that the transgene expression was not associated with accumulation of aberrant levels of galectin-3. Adhesion of galectin-3 expressing Σ12 cells on laminin had a 2.5-fold decrease, as compared to the control transfectants (Fig. 3E). Concomitantly, galectin-3 expressing Σ12 cells were more migratory in response to laminin-111 than the parental cells (Fig. 3E). The migratory response was clearly dependent on extracellular galectin-3, since it could be inhibited by exogenous lactose, but not sucrose (Fig. 3E). Transient transfection of galectin-3 gene in S11 cells also led to an increase in their migratory response to laminin-111 (Fig. 3F). To evaluate the role of extracellular galectin-3 in the migratory response of sarcoma cells, migration of galectin-3 null Σ12 cells was studied either in the absence or presence of exogenous recombinant galectin-3 (Fig. 3G). The migratory response of sarcoma cells to extracellular galectin-3 was dose-dependent (Fig. S6). Laminin-111 induced migration was evaluated using the scrape assay. In the absence of galectin-3, differences in migration were not statistically significant. A robust and significant increase in cell migration was observed when cells were exposed to galectin-3. This increase was inhibited in the presence of lactose, reconfirming the notion that the effect of galectin-3 on cell migration depended upon its lectin activity.

### Extracellular galectin-3 induced the disassembly of focal adhesion plaques

**Figure 4:**
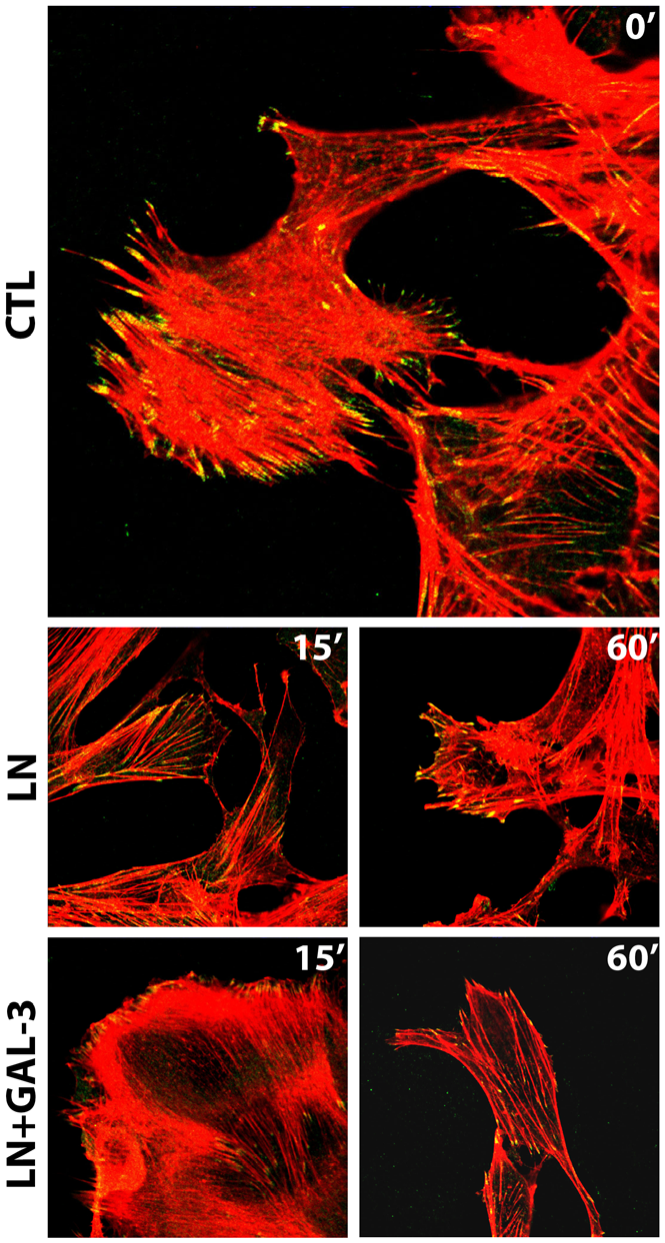
Extracellular galectin-3 (Gal-3) promotes the disassembly of stable focal adhesion plaques by decreasing the amount of phosphorylated FAK in the lamellipodia of migrating cells. Σ12 cells were grown on coverslips and were subjected to the scrape assay either in the absence (ctl) or presence of laminin-111 (LN) or laminin-111 and 20 µg/mL galectin-3 (LN+gal-3) for 15 minutes. Cells were either fixed after 15 minutes after the migration stimulus. Confocal photomicrographs of typical fields are shown. Intracellular distribution of phosphorylated FAK (green) and organization of stress fibers (phalloidin staining, in red) are shown. Control cells showed mature adhesion plaques, as shown by staining of phosphorylated FAK. In the presence of galectin-3, there was a fast disassembly of the adhesion plaques indicated by the decrease of phosphorylated FAK in the lamellipodia.

Cell migration is a complex process that depends on a precisely orchestrated series of events involving adhesion, deadhesion and rearrangements of the cytoskeleton. Signals are processed at focal contact points, supramolecular structures formed upon ligation and clustering of integrins followed by the recruitment of specific proteins [32], [33]. To investigate the mechanisms underlying galectin-3-induced cell migration, we examined the levels of tyrosine phosphorylation of proteins involved in focal adhesion turnover. We analyzed the effects of extracellular galectin-3 on the organization of focal adhesion plaques in the galectin-3^−/−^ Σ12 cells. In stationary cells, focal adhesion plaques containing phosphorylated FAK form organized stress fibers (Fig. 4, ctl panels and Fig. S4). When cells are stimulated to migrate, these structures are disassembled and the cytoskeleton is reorganized, thus allowing cell movement. Using the scrape assay, we followed laminin-induced cell migration either in the absence (LN) or presence of galectin-3 (LN+gal-3) for 15 minutes, as illustrated in Fig. 4. Exposure of cells to extracellular galectin-3 resulted in a significant decrease in the amount of phosphorylated FAK in cell protrusions, followed by an extensive reorganization of cytoskeletal actin and membrane ruffling as compared to control conditions (ctl or LN) or cells exposed to galectin-3 in the presence of lactose (LN+gal-3+lac) (Fig. S4).

**Figure 5:**
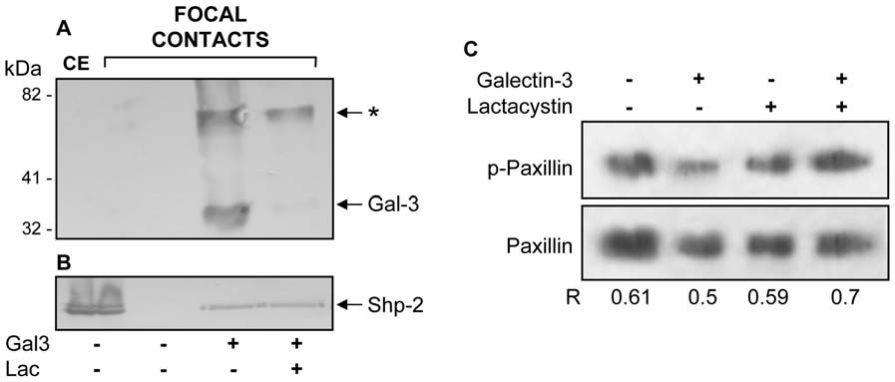
Analysis of proteins enriched in the focal complexes in the absence (-) or presence (+) of extracellular galectin-3. Σ12 cells were exposed to 10 µg/mL laminin and 20 µg/mL galectin-3 for 15 minutes and focal complexes were then prepared and analyzed regarding the presence of galectin-3 (**A**), SHP-2 (**B**) and phospho-paxillin (**C**) using Western blot analysis. (**Panel A**) Recruitment of galectin-3 to focal complexes was inhibited in the presence of lactose (lane at the right in panel A). Two bands were recognized by M3/38 monoclonal antibody in focal adhesion extracts; one which migrates with an apparent molecular weight of 30–35 kDa (Gal-3) and a second one which migrates as a 70 kDa-band (arrow). CE, in the left lane in panels A and B refer to Σ12 cellular extracts. (**Panels B and C**) Exposure of Σ12 cells to extracellular galectin-3 led to an increase in SHP-2 (panel B) and decrease of phosphorylated paxillin in the focal complexes (panel C). Prior inhibition of proteasome with lactacystin abrogated the decrease of phosphorylated paxillin in focal complexes. Representative blot of paxillin and phosphorylated paxillin are shown and the densitometric analysis of two independent experiments were performed, yielding similar results.

The Src homology 2 domain-containing protein tyrosine phosphatases SHP-2 dephosphorylate FAK and paxillin [34], [35]. The decrease in the amount of phosphorylated FAK in the lamellipodia of migrating cells exposed to extracellular galectin-3 for 15 minutes was associated with the recruitment of the tyrosine phosphatase Shp-2 to the focal complexes, as shown in Fig. 5B. Paxillin is another important focal adhesion molecule that is associated with and phosporylated by FAK [36], [37]. To explore whether paxillin phosphorylation was also regulated by galectin-3 treatment, phospho-paxillin levels were measured after exposure of galectin-3. As shown in Figure 5C, as seen for FAK phosphorylation (Fig. 4), galectin-3 mediated a decrease in paxillin phosphorylation levels. Intriguingly, in the presence of the proteasome inhibitor lactacystin, there was an increase in the amount of phosphopaxillin after exposure of cells to galectin-3.

### Galectin-3 activates PI-3 kinase signaling

**Figure 6:**
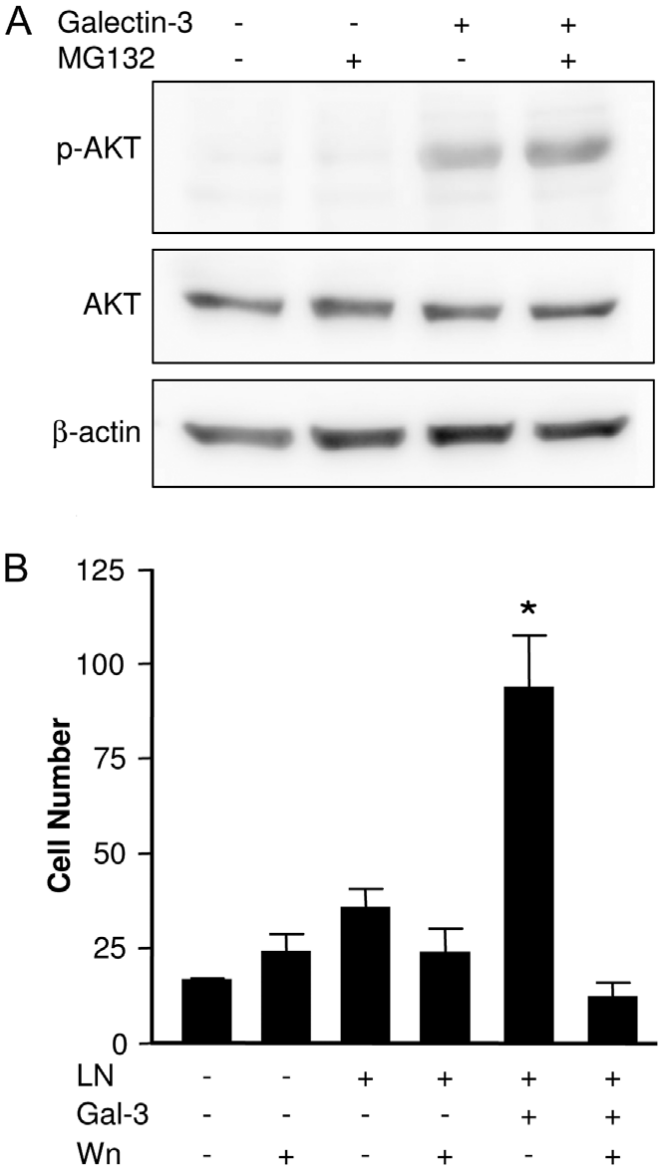
Galectin-3 increases Σ12 cell motility through a PI-3 kinase dependent pathway. (**A**) Σ12 cells were exposed to 10 µg/mL laminin-111 either in the absence or presence of 20 µg/mL galectin-3 and of the proteasome inhibitor MG-132 for 15 minutes and phospho-AKT was analyzed using Western blot analysis. Extracellular galectin-3 leads to increase of phosphorylated AKT. Representative blot of AKT and phosphorylated AKT are shown and the densitometric analysis of two independent experiments were performed. (**B**) Σ12 cells were grown in coverslips and subjected to the scrape assay. Migration of Σ12 cells into the scratched area was measured after 24 hours by direct counting of DAPI-stained cells using a graticle projected onto micrographs collected using a fluorescence microscope. Cells were incubated or not in the presence of 10 µg/mL laminin-111 (LN), 20 µg/mL galectin-3 (Gal-3) and 1 mM wortmannin (Wn), as indicated. Bars represent mean of three independent assays done in triplicates, the SD is also represented. Analysis was done using one-way ANOVA, followed by the Bonferroni's *t*-test for multiple comparisons. There were no statistically significant differences among the groups treated in the absence of galectin-3. Exposure of Σ12 cells to galectin-3 and laminin-111 increased their motility about 2.5 fold over control conditions and over exposure of laminin-111 alone (*p*<0.001). The galectin-3 effect was inhibited by wortmannin (*, *p*<0.001), which did not affect significantly the migratory capacity of cells under the other experimental conditions.

In addition to acting as an adaptor protein, interacting and recruiting multiple target proteins to focal adhesion complexes, FAK modulates signaling pathways as those triggered by the Rho family, which coordinates cellular processes such as adhesion, migration and actin cytoskeleton reorganization [38]. Rac1 is a member of the Rho family of small GTP-ases and plays a fundamental role in actin cytoskeleton reorganization, lamellipodia formation, and cell migration [38]. Rac1 interacts with specific effectors, including p21 activated kinases (PAK) [39]. Exposure of corneal epithelial cells to galectin-3-activated Rac1, increasing their migratory capacity [40]. To determine whether galectin-3 induced the activation of Rac1-dependent signaling pathways in sarcoma cells, we evaluated whether phosphorylated PAK accumulated in focal contacts upon exposure to galectin-3. PAK was not found in focal contacts and remained in the cytoplasm of Σ12 cells exposed to galectin-3 (Fig. S5). Alternatively, it was shown that Rac1 can activate PI-3 kinase signaling in epithelial cells and its activation is sufficient to disrupt epithelial polarization and induces cell motility and invasion [41]. Migrating cells exposed to galectin-3 showed increased AKT phosphorylation levels (Fig. 6A) and inhibition of PI-3 kinase dependent pathway using wortmannin abolished the promigratory activity of galectin-3 (Fig.6B), indicating that galectin-3 acts as a positive modulator of laminin-111-induced cell migration via a PI-3 kinase dependent pathway.

## Discussion

Here we have provided evidence for the involvement of extracellular galectin-3 as a positive modulator of sarcoma cell migration on laminin-111. *De novo* expression of galectin-3 in a galectin-3^−/−^ sarcoma cell line decreased its adhesion and promoted its migratory capacity on laminin-111, in a carbohydrate-dependent manner. Modulation of migration was associated with the recruitment of Shp2 tyrosine phosphatase to focal complexes and an apparent acceleration of FAK and paxillin turnover on focal contacts, as evaluated by the decrease of phosphorylated-FAK in lamellipodia of migrating cells and paxillin in focal complexs. Migrating sarcoma cells presented increased phosphorylation levels of AKT and the promigratory activity of galectin-3 was inhibited by wortmannin, indicating its dependence on the activation of PI-3 kinase pathways.

Accumulation of galectin-3 upon transformation of cells that do not express this lectin under physiological conditions has been observed in different models of tumor progression [42], [43]. Accumulation of galectin-3 is associated with malignant transformation of fibroblasts and other mesenchyme-derived cells [43]–[45]. Khanna and colleagues (2001) have shown that galectin-3 accumulates in more metastatic osteosarcoma cells, thus providing an association between galectin-3 expression and a more invasive phenotype. In sarcoma cells, such as CCR2 cells which express high amounts of galectin-3, migrating cells display a significant amount of the lectin in the lamellipodia, as illustrated in Fig. 1. Galectin-3 accumulation in different cell types within specialized tissue microenvironments have been associated with the acquisition of a migratory phenotype. We and others showed that galectin-3 was found overexpressed in glioblastomas [23], [46], especially within pseudopalisades, which are formed by migrating cells surrounding necrotic areas [47]. In the thymus, galectin-3 is produced by stromal cells. We have previously shown that galectin-3 could impair integrin-dependent intercellular interactions between thymocytes and thymic epithelial cells [48], leading to migration of CD4^+^CD8^+^ thymocytes [49]. Galectin-3 modulates the formation of focal contacts composed by laminin-332, α3β1 and α6β4 integrins promoting keratinocyte motility [50]. Taken together, these data strongly suggest that extracellular galectin-3 plays an important role in the control of adhesion and migration.

The sarcoma cell lines used in this study (S11 and S12) express galectin-3 and the protein is displayed on the cell surface (Fig. 2B and 2C). Despite the lack of a classical signal peptide for secretion, galectin-3 can be transferred to the extracellular space, via a non-classical pathway [51], [52]. The ability of galectin-3 to cross the lipid bilayer of large unilamellar vesicles suggests that this lectin has an yet to be identified signal sequence, or posttranslational modification, that enables it to interact and traverse the plasma membrane [53]. Once secreted, galectin-3 interacts with extracellular matrix proteins/glycoproteins and their receptors [40], [54]–[56]. Exposure of cells to galectin-3 ligands, such as fetuin, induced secretion of galectin-3 [57]. Since laminin-111 is also a galectin-3 ligand, it is conceivable that exposure of cells to laminin would induce local secretion of galectin-3. Recruitment of galectin-3 to the lamellipodia could be a first step towards its vectorial secretion, as illustrated in Fig. 1. Besides secretion, galectin-3 could be released into the extracellular space following cell lysis, which does occur in tumor microenvironments, for example. Release [57] of galectin-3 from necrotic cells may have an impact on tissue repair, as it is a potent chemoattractant for leukocytes [16], [58] and some epithelial cells [59]. Most chemoattractants lead to the inside-out activation of integrins [60]. In the case of galectins, integrins are also putative ligands for galectin-3, suggesting the existence of an alternative outside-in pathway that could lead to modulation of the integrin function [40], [61].

Our results showed that extracellular galectin-3 led to a de-adhesion state, what has been shown in different cellular models *in vitro* [48], [55], [62] and *in vivo* [40], [49]. Considering the possibility of a polarized secretion of galectin-3 at the cell leading edge, this de-adhesive state could contribute to cell migration, as galectin-3 led to the destabilization of focal complexes. We also showed that galectin-3 led to the recruitment of SHP-2 tyrosine phosphatase and to a decrease in the amount of phosphorylated FAK in focal adhesion plaques, suggesting an acceleration of the turnover of FAK in these structures. Recent elegant studies have implicated galectin-3 in the disassembly of focal adhesion complex [32], [40], [63], [64], however in the models studied the absence of a galectin-3 induced lattice was associated with reduced phosphorylation of FAK. It is important to point out that the role of FAK in promoting either cell motility or invasion is through the activation of different signaling pathways [65], therefore it may not be possible to predict just by the state of phosphorylation of FAK whether cells would adhere strongly or migrate. FAK has been implicated in growth-factor-induced cell motility through dephosphorylation and inactivation upon stimulation of growth factor receptors in a variety of human tumor cells, suggesting that dephosphorylation of adhesion-related proteins may be a common event associated with tumor migration and invasion. EGF and IGF-1-receptor induced migration and invasion is also associated with dephosphorylation of FAK, p130^cas^ and paxillin [66]. In agreement with the phenomenon observed in sarcoma cells, Pierce and colleagues showed that knocking down of MGAT5 expression, which led to decreased synthesis of galectin-3 ligands in molecules such as the EGFR, interfered with the migratory response of breast cancer cells in response to EGF. The EGF-induced migratory phenotype is associated with activated SHP-2, dephosphorylation of FAK and accelerated turnover of focal adhesion contacts [67]. We have also observed a decrease in the amount of phosphorylated paxillin upon exposure of sarcoma cells to galectin-3. In this particular case, decreased amounts of phosphorylated paxillin was associated with proteasomal activity, as treatment of cells with lactacystin restored the amount of phosphorylated paxillin to control levels. Regardless, exogenous galectin-3 led to decreased phosphorylation of FAK and loss of phosphorylated paxillin from the focal adhesion complexes, favoring sarcoma cell migration. Interestingly, cell adhesion to other matricellular proteins, such as tenascin and SPARC, also triggered paxillin tyrosine dephosphorylation [68].

FAK can trigger activation of small GTPases, such as Rac and Cdc42, that regulate extension of filopodia and lamellipodia at the leading edge and the formation of focal adhesion complexes during cell migration [38]. The best characterized effector of Rac and Cdc42 that mediates cell motility is p21-activated kinase (PAK) [38]. Galectin-3 does not lead to PAK phosphorylation and recruitment to focal adhesion complexes, suggesting the involvement of another signaling pathway in the migratory response of sarcoma cells to galectin-3. There is evidence that Rac and Cdc42 can regulate cell migration through activation of the PI-3 kinase signaling pathway [41], [69]. This is the case of the sarcoma cells studied herein, since exposure to galectin-3 led to a significant increase in phospho-AKT levels and wortmannin inhibited the promigratory activity of galectin-3. Likewise, in other mesenchymal cells, such as smooth muscle cells, IGF-1-triggered migration depends on the activation of SHP-2, which in turn leads to the activation of PI-3 kinase [70].

Altogether, our results indicate that extracellular galectin-3 acts as a matricellular protein [71], like tenascin-C [72], thrombospondin [73], SPARC [74], and galectin-8 [75]. It is tempting to speculate that through its binding to integrins or integrin-associated molecules, galectin-3 interferes with the structural organization of platforms containing the ECM receptors. As it is demonstrated, galectin-3 tends to oligomerize when bound to its ligands [76], therefore galectin-3 and their ligands may form a lattice on the cell surface, as suggested by Dennis and colleagues [77]–[79]. This lattice may justify the enrichment of galectin-3 on focal adhesion complexes, as we have observed in Σ12 cells exposed to exogenous galectin-3. The ratio between the amount of extracellular galectin-3 and their ligands in the extracellular matrix and on the cell surface dictates the structure of this molecular lattice. Density of signal transducing molecules in very close proximity within the supramolecular structure of these lattices would determine cell responses, such as adhesion or migration. Identification and recruitment dynamics of the proteins present in these supramolecular structures is now warranted.

## Materials and Methods

### Ethics Statement

All animal work conducted during this project was approved by the Ethics Committee of Faculdade de Medicina da Universidade de São Paulo, which followed the guidelines of the American Association of Veterinary Medicine. All animal manipulations were performed by trained personnel. Tumor bearing animals were euthanized when the larger tumor diameter reached 1 cm, using IP injection of anesthetics.

### Reagents

Unless otherwise stated, reagents were purchased from Sigma (St. Louis, MO). Rat anti-α6 GoH3 was purchased from Coulter (Marseille, Fr) and FITC-conjugated anti-CD29 was from Pharmigen (San Diego, CA). Mouse anti-P-FAK was purchased from Chemicon (Temecula, CA). Rabbit anti-FAK and anti-SHP2 antibodies were purchased from Santa Cruz Biotechnology, INC (Santa Cruz, CA). Rabbit anti-mouse AKT and anti-P-AKT were purchased from Cell Signaling Technologies (Danver,MA). Rabbit anti-rat IgG horseradish peroxidase-conjugated antibodies were purchased from KPL- Kirkegaard & Perry Laboratories (Gaithersburg, MD). Laminin-111 was kindly provided by Dr. Vilma Martins (Ludwig Institute for Cancer Research, São Paulo).

### Animals

Both galectin-3^−/−^ (Gal-3^−/−^) and wild type C57bl/6 (Gal-3^+/+^) mice came from F.-T. Liu's laboratory and were generated as described elsewhere [80]. Mice were housed under conventional conditions approved by the University of São Paulo-Medical School Ethics committee and local animal research facility. Six to eight week old animals were used in all the experiments.

### Induction of sarcomas

Both wild type and galectin-3^−/−^ mice were given two subcutaneous injections of 50 µg of methylcholanthrene in tricapriylic oil. The first dose was given to 6–8 week old mice, the second dose was given twelve weeks after and the development of tumors was then followed weekly. When tumors reached 0.5–1 cm^3^, mice were sacrificed under aseptic conditions, and tumors were dissected, minced into small fragments (0.01–0.05 cm^3^), which were maintained in culture. Part of tumor fragments were fixed in phosphate-buffered paraformaldehyde and then processed for routine histopathology.

### Cells and Culture conditions

The murine cell lines S11, S12 e Σ12 were established from the explanted methylcholanthrene-induced sarcomas. The Σ12 cell line was derived from a galectin-3^−/−^ background, while both S11 and S12 cell lines were derived from the wild type genotype. The CCR2 cell line was established by transformation of NIH3T3 fibroblasts with an activated form of EJ-*ras*, as described elsewhere [4]. The hybridoma TIB-166/M3-38 (American Tissue Culture Collection), which secretes a rat monoclonal antibody against galectin-3, was cultured in RPMI 1640 supplemented with 20% fetal calf serum.

### Transient transfection

S11 and Σ12 cells were transiently transfected with either pEf1neo or pEf1neo-gal3 using Lipofectamine™ (GIBCO BRL, Grand Island, NY), according to the manufacturer's protocol. After 24–72 h, transfected cells were checked for the expression of the transgene by western blotting and used in the assays described below. Transfection efficiency was typically around 15% of cells.

### Western blotting

Protein extracts were prepared using lysis buffer (1% Triton X−100 in 150 mM NaCl and 50 mM Tris pH7.4 , containing 5 mM EDTA, 2 µg/mL aprotinin and 1 mM PMSF) and kept at −20°C until use. Aliquots of 10–40 µg of proteins were boiled in SDS-sample buffer (240 mM Tris-HCl pH 6.8, 0.8% SDS, 200 mM β-mercaptoethanol, 40% glycerol and 0.02% bromophenol blue) for 5 min and then separated by electrophoresis in 12.5% SDS-polyacrylamide gels and transferred to a polyvinylidene difluoride membrane (Millipore Corp., Bedford, USA). Protein loading was always confirmed in a second gel run simultaneously and stained with Coomassie blue. After protein transfer, the membranes were blocked with 5% nonfat dry milk in PBS (10 mM phosphate buffer pH 7.2, 150 mM NaCl), incubated with the indicated antibodies overnight at 4°C, followed by incubation with appropriate secondary antibodies and development with either diaminobenzidine, chemiluminescence assay or further incubation with ^125^I-protein A.

### Flow cytometry analysis

Cultured cells were harvested with trypsin and EDTA. After trypsin inactivation, cells were washed with PBS containing 1% BSA and incubated with different antibodies. Typically, 10^6^ cells were incubated at a final concentration of 2.5 to 4 µg/mL of anti-integrins or anti-galectin-3 for 1 hour at 4°C. After 3 washes with PBS containing 0.1% BSA, cells were incubated with 1 µg/mL FITC-conjugated secondary antibodies for 1 hour at 4°C. After washing, cells were analyzed in a flow cytometer (FACScalibur, Becton Dickinson).

### Migration assay

Migration assays were performed in Transwell chambers (Costar, NY) essentially as described elsewhere [4]. Briefly, a 10 µg/mL solution of laminin-111 in DMEM was used to coat the lower surface of the polycarbonate filter for 2 hours at 37°C. The filter was then blocked with DMEM containing 1% BSA for 1 hour. After harvesting, 2x10^5^ cells were suspended in 0.5 mL DMEM and added in the upper compartment of the Transwell chambers, either in the absence or presence of indicated concentrations of lactose or sucrose for 2 to 4 hours at 37°C. Filters were then washed with PBS, fixed in 3.7% formaldehyde for 15 min, stained with toluidine blue for 5 min. Non-migrating cells present in the upper compartment were removed with a cotton swab. The migrating cells were counted directly, using an inverted microscope. Every assay point was done in triplicates in three independent experiments. At least ten distinct medium power fields (objective x20), corresponding to a graticle-defined area of 0.25 mm^2^, were counted for each filter. Analysis was done using ANOVA followed by the Bonferroni's *t* test for multiple comparisons, using the GraphPad Prism software.

### Cell adhesion

The adhesion assay was performed essentially as described elsewhere [7]. Briefly, 24-well cell culture clusters (Costar, NY) were coated with increasing concentrations of laminin-111 in DMEM overnight at 4°C. The plate was then blocked with 2% BSA in PBS for 1 h at 37°C and washed three times with PBS. Cells were suspended in DMEM without serum and added to each well (10^5^ cells/well). After two hours at 37°C, non-adherent cells were washed out gently. Adherent cells were then fixed, stained as above and counted directly using an inverted microscope, as was done for migration assays.

### Preparation of galectin-3

Recombinant human galectin-3 was produced in *E. coli* and purified by affinity chromatography on lactosyl-agarose as described previously [81], followed by extensive dialysis against PBS pH 7.4. Galectin-3 was then concentrated by ultradiafiltration using Amicon Ultra-15 centrifugal filters (Millipore, Bedford, MA). Galectin-3 concentration was determined by using the Bio-Rad Dc Protein Assay (Bio-Rad Laboratories, Hercules, CA), separated into aliquots, and stored at −20°C until use.

### Effects of galectin-3 on the distribution of focal contact proteins

Σ12 cells were grown in DMEM supplemented with 10% FBS on glass coverlips to near confluence for 48 h. After 2 washes with PBS, the cells were subjected to the scrape assay. A wound was made and the cells were incubated with 10 µg/mL laminin-111 in the absence or presence of 20 µg/mL galectin-3, either in the presence or absence of 100 mM lactose, for 15 minutes in medium under serum-free conditions at 37°C and then fixed and processed for immunofluorescence staining. Alternatively, 5 million Σ12 cells were seeded in 100 mm diameter tissue culture plates and exposed or not to 10 µg/mL laminin-111 and 20 µg/mL extracellular galectin-3 for 15 minutes. Alternatively, Σ12 cells were treated or not with 20 µg/mL MG132 or 5.3 µg/mL Lactascystin for four hours and after exposed or not to 10 µg/mL laminin-111 and 20 µg/mL extracellular galectin-3 for 15 minutes. After these time periods, focal adhesion plaques were isolated following the protocol proposed by Avnur and Geiger [82]. Briefly, cells were washed twice in the washing buffer (5.5 mM glucose, 5.4 mM KCl, 137 mM NaCl, 0.4 mM KH_2_PO_4_, 0.4 mM Na_2_HPO_4_.H_2_O, 4 mM NaHCO_3_, 3.2 mM PIPES, 1.9 mM MgCl_2_ and 0.5 mM CaCl_2_, pH 6.1), and then incubated with 0.2% saponin in the washing buffer for 5 min at room temperature. Cell bodies were then removed by gently flushing with the washing buffer using a syringe. After removal of all cell bodies, controlled by direct visualization using an inverted microscope, the extraction buffer (8 M urea, 1 mM Tween 20 and 100 mM DTT) was then added to the plate. After 5 minutes, the extract was collected and kept frozen at −20°C until use.

### Scrape assay

Cells were grown on glass coverslips until confluence when a scratch wound was made [83]. Cells were then washed and maintained in culture for 15 minutes in serum-free medium, in the presence of exogenous laminin-111 (10 µg/mL) and galectin-3 (20 µg/mL) either in the presence of lactose (100 mM) or wortmannin (1 mM) as indicated in the results session. The cells were then fixed with 1% paraformaldheyde in PBS for 15 min, permeabilized with 0.03% saponin in PBS for 15 min, and then processed according to the aim of the assay. For experiments aiming at quantifying migrating cells into the wounded area, cell nuclei were stained with DAPI and counted using a fluorescence microscope, essentially as described above. Relative migration was calculated for each experimental group. Analysis was performed using one-way ANOVA, followed by the Bonferroni's *t* test for multiple comparisons. For assays aiming at the analysis of focal adhesion plaques, fixed cells were incubated with 1% BSA in PBS for 1 hour in order to block nonspecific interactions with immunoconjugates. The cells were then incubated with the indicated antibodies and suitable fluorochrome-conjugated secondary antibodies or with rhodamine-conjugated phalloidin. Coverslips were mounted on slides using a solution containing glycerol and anti-fading agents and analyzed by either conventional fluorescence or confocal microscopy, as indicated. For FAK staining, the fixation buffer contained 4% paraformaldehyde in 0.1 M phosphate buffer, pH 7.4 containing 10 mM PIPES, 5 mM EGTA, 2 mM MgCl_2_ and 0.1% Triton X−100. For galectin-3 staining was used the TIB-166/M3–38 antibody. Cells were fixed at room temperature for 15 min, washed and then post-fixed with 95% ethanol at –20°C for 5 min.

## Supporting Information

Figure S1 **Histopathological analysis showed that tumors from wild type** (**A**) **and galectin-3^−/−^ mice** (**B**) **were sarcomas.**
(TIF)

Figure S2 **S11, S12 and Σ12 cells display a similar pattern of α6 and β1 integrin, the sarcoma laminin binding integrin chains, on cell surface.** Levels of laminin-111 binding integrin on S11, S12 and Σ12 cell surface were determined by flow cytometry. α6 and β1 integrin levels were similar in all three cell lines.
(TIF)

Figure S3 **Galectin-3 expression does not alter the expression pattern of laminin binding integrins on Σ12 cell surface.** Σ12 cells were transiently transfected with the pEf1neo (Neo) and with pEf1neo-gal-3 (Neo-Gal-3) plasmids. After 48 hours of transfection, cells were harvested and analyzed regarding surface expression of both α6 and β1 integrin chains using flow cytometry. No significant differences were observed in the integrin levels in the transfected cells.
(TIF)

Figure S4 **Extracellular galectin-3** (**Gal-3**) **decreases the amount of phosphorylated FAK followed by extensive reorganization of actin fibers and membrane ruffling.** The effect of exogenous galectin-3 (gal-3) on LN-induced migration was followed either in absence or presence of lactose (LN+gal-3 and LN+gal-3+lac, respectively) for 15 minutes. The decrease of phosphorylated FAK (staining in green) and stress fibers (phalloidin staining in red) in focal contacts by galectin-3 is partially reverted in the presence of lactose as indicated by phalloidin staining in red.
(TIF)

Figure S5 **PAK is not recruited to focal adhesions in the presence of galectin-3.** Σ12 cells were grown on coverslips and were subjected to the scrape assay either in the absence (ctl) or presence of laminin-111 (LN). The effect of exogenous galectin-3 (gal-3) on LN-induced migration was followed (LN+gal-3). Cells were fixed for 15 minutes after migration stimulus. Confocal photomicrographs of typical fields are shown intracellular phosphorylated PAK distribution stained in green and actin filaments in red (labeling with rhodamine-conjugated phalloidine).
(TIF)

Figure S6 **Extracellular galectin-3 increases Σ12 cell migration in a dose-dependent manner.** Σ12 cells were grown in coverslips and subjected to the scrape assay. Migration of Σ12 cells into the scratched area was measured after 24 hours by direct counting of DAPI-stained cells using a graticle projected onto micrographs collected using a microscope. Cells were incubated or not in the presence of 10 µg/mL laminin-111 (LN) and 5, 10 or 20 µg/mL galectin-3 (Gal-3), as indicated. Results are means of two independent experiments performed in triplicate with p<0.05 when compared migrating cells in the presence of 5 and 10 µg/mL galectin-3 and p<0.0001 when compared migrating cells in the presence of 20 µg/mL galectin-3 with control cells.
(TIF)
