# Supplementary figures and images for: The Promigratory Activity of the Matricellular Protein Galectin-3 Depends on the Activation of PI-3 Kinase
Source: PLoS One. 2011 Dec 28;6(12):e29313. doi: 10.1371/journal.pone.0029313 (PMC3247242; doi:10.1371/journal.pone.0029313)

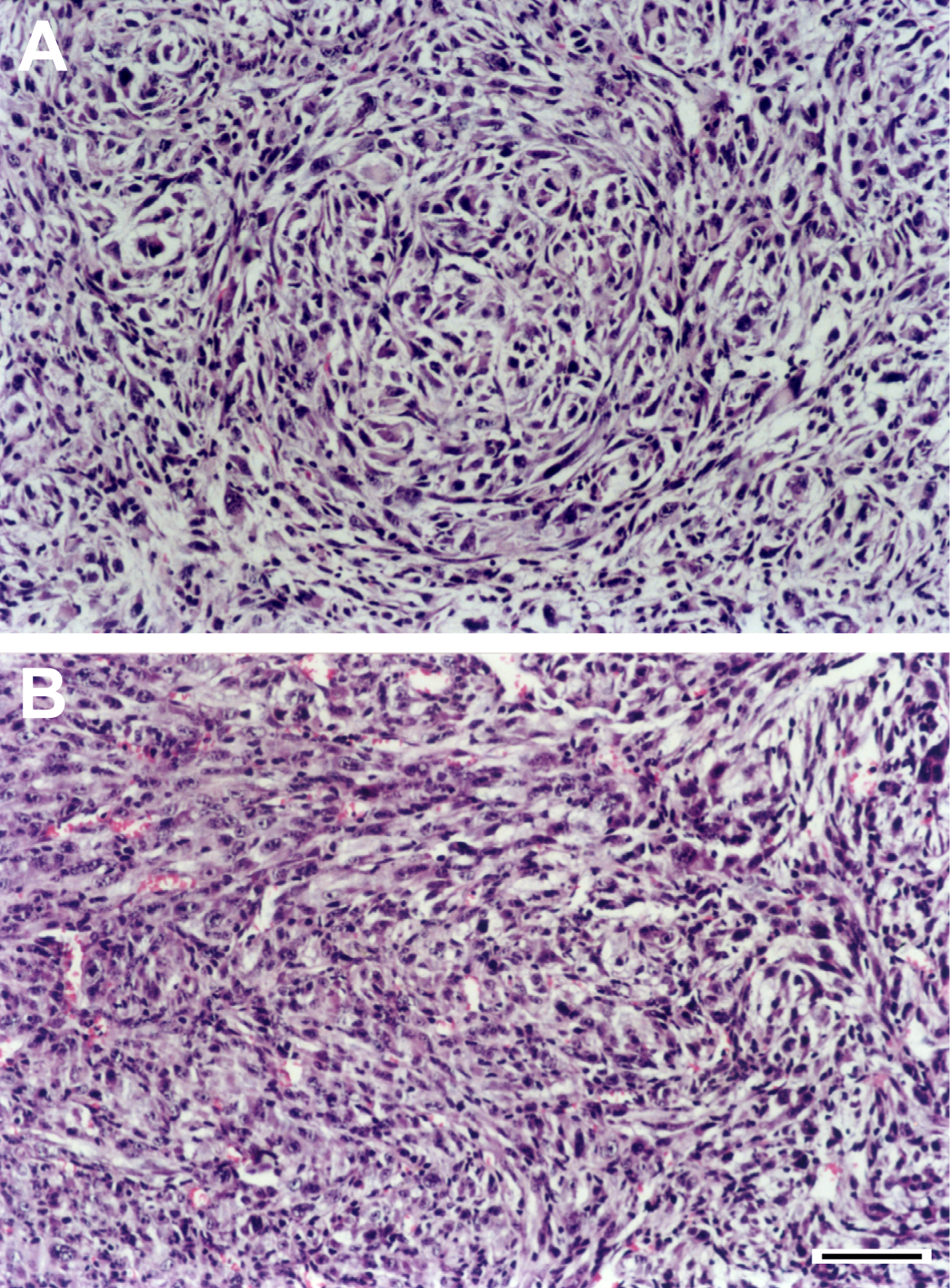

Supplement: Figure S1 — Histopathological analysis showed that tumors from wild type (A) and galectin-3−/− mice (B) were sarcomas. (TIF) [file pone.0029313.s001.tif]

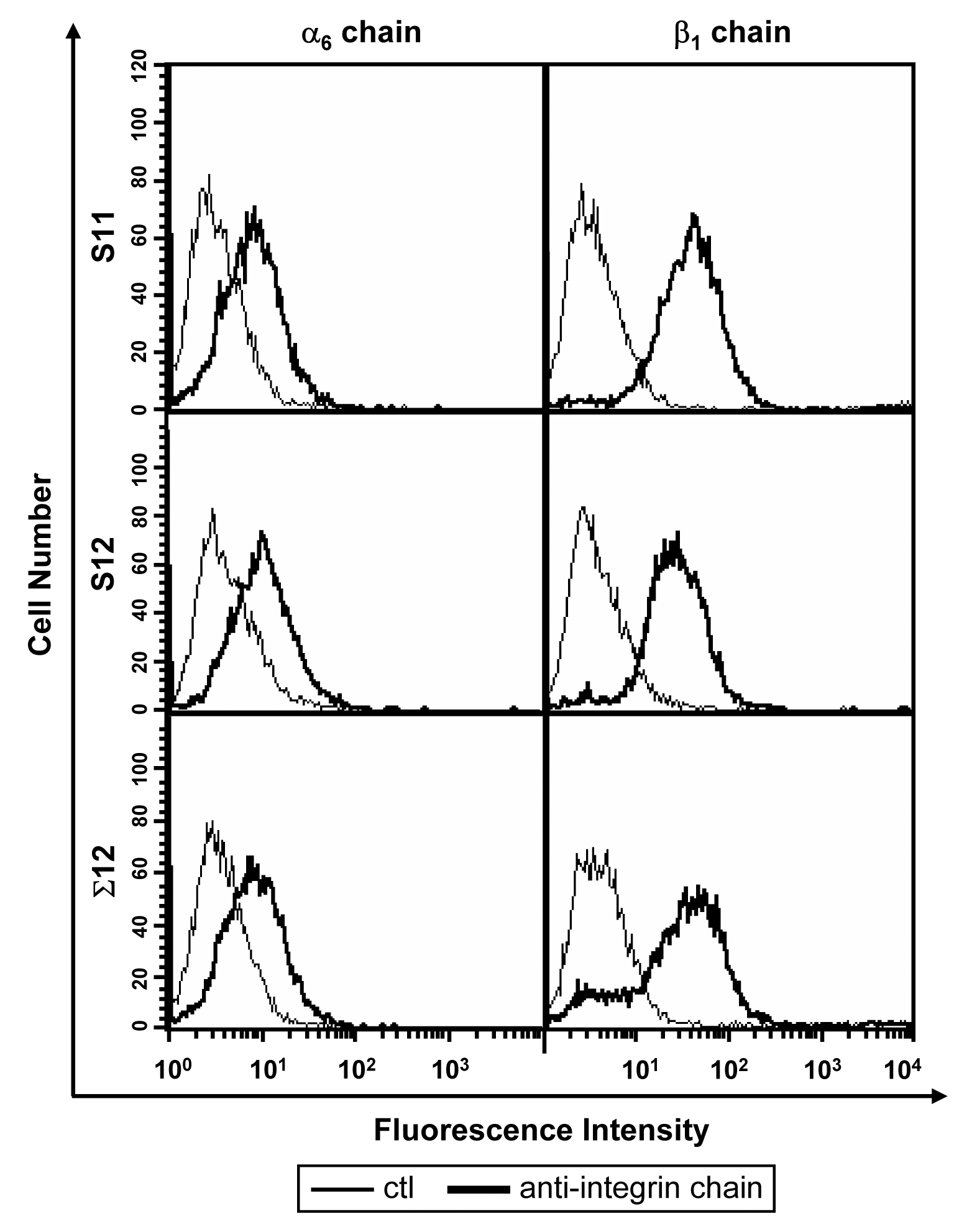

Supplement: Figure S2 — S11, S12 and Σ12 cells display a similar pattern of α6 and β1 integrin, the sarcoma laminin binding integrin chains, on cell surface. Levels of laminin-111 binding integrin on S11, S12 and Σ12 cell surface were determined by flow cytometry. α6 and β1 integrin levels were similar in all three cell lines. (TIF) [file pone.0029313.s002.tif]

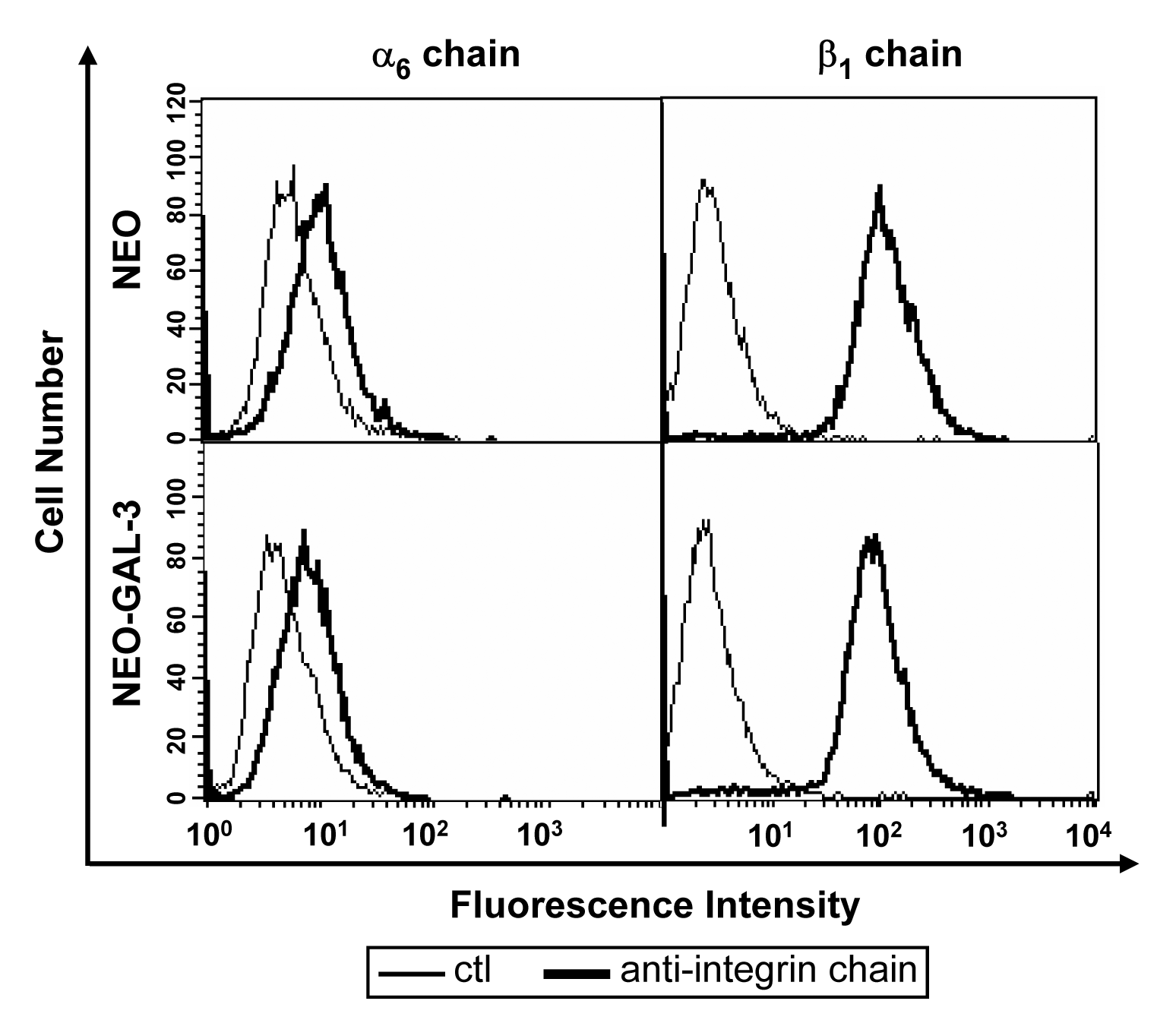

Supplement: Figure S3 — Galectin-3 expression does not alter the expression pattern of laminin binding integrins on Σ12 cell surface. Σ12 cells were transiently transfected with the pEf1neo (Neo) and with pEf1neo-gal-3 (Neo-Gal-3) plasmids. After 48 hours of transfection, cells were harvested and analyzed regarding surface expression of both α6 and β1 integrin chains using flow cytometry. No significant differences were observed in the integrin levels in the transfected cells. (TIF) [file pone.0029313.s003.tif]

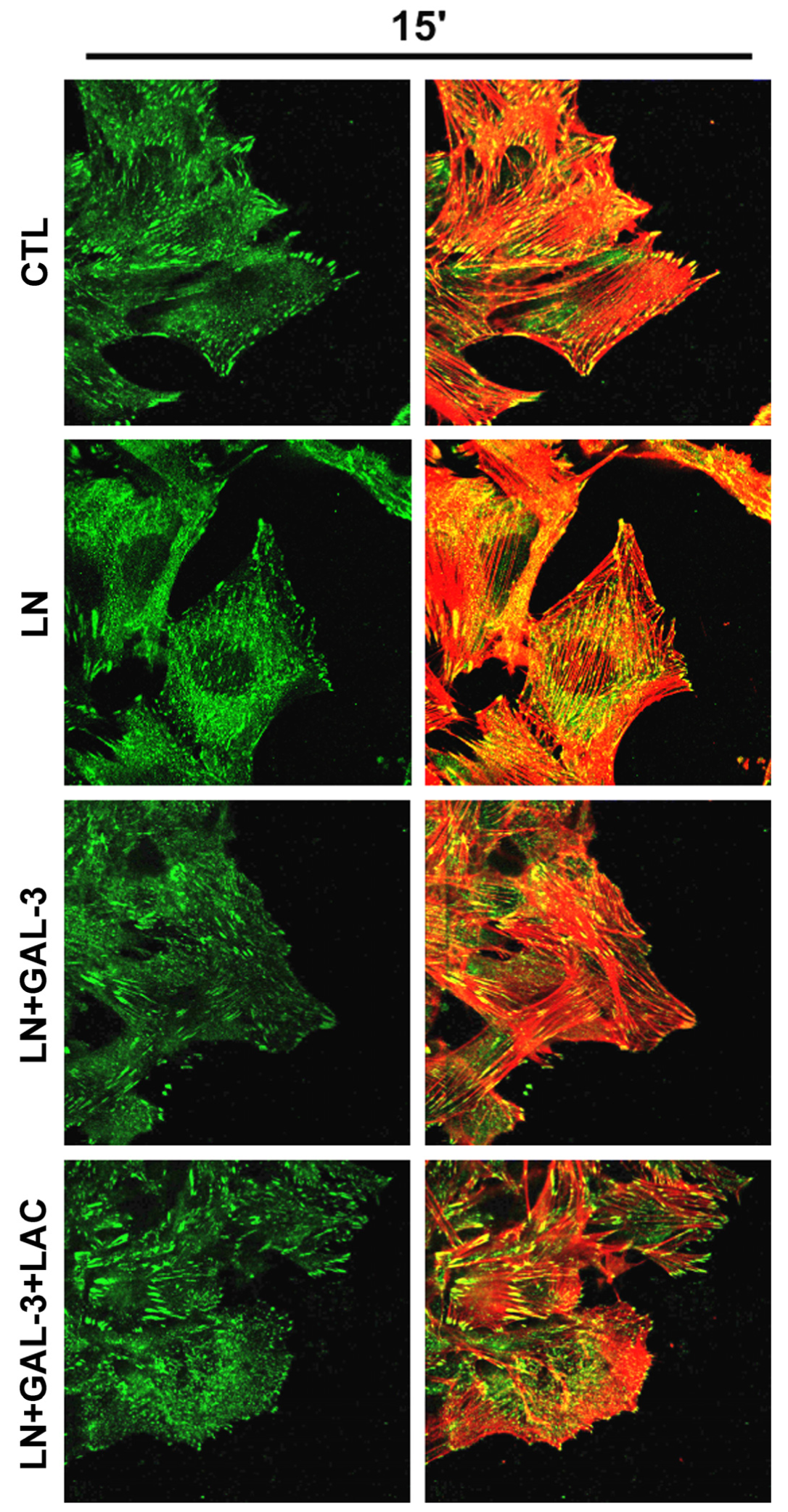

Supplement: Figure S4 — Extracellular galectin-3 (Gal-3) decreases the amount of phosphorylated FAK followed by extensive reorganization of actin fibers and membrane ruffling. The effect of exogenous galectin-3 (gal-3) on LN-induced migration was followed either in absence or presence of lactose (LN+gal-3 and LN+gal-3+lac, respectively) for 15 minutes. The decrease of phosphorylated FAK (staining in green) and stress fibers (phalloidin staining in red) in focal contacts by galectin-3 is partially reverted in the presence of lactose as indicated by phalloidin staining in red. (TIF) [file pone.0029313.s004.tif]

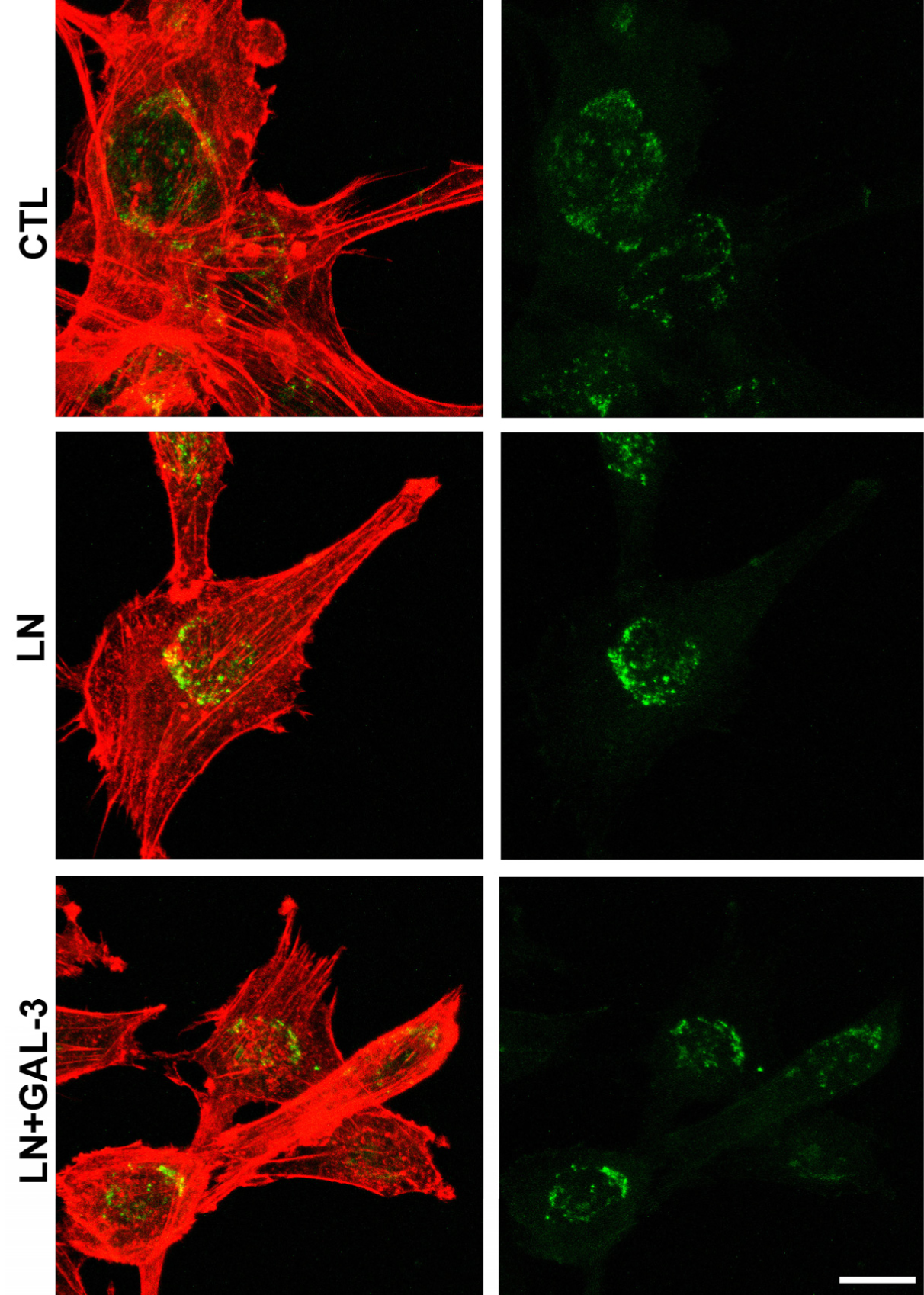

Supplement: Figure S5 — PAK is not recruited to focal adhesions in the presence of galectin-3. Σ12 cells were grown on coverslips and were subjected to the scrape assay either in the absence (ctl) or presence of laminin-111 (LN). The effect of exogenous galectin-3 (gal-3) on LN-induced migration was followed (LN+gal-3). Cells were fixed for 15 minutes after migration stimulus. Confocal photomicrographs of typical fields are shown intracellular phosphorylated PAK distribution stained in green and actin filaments in red (labeling with rhodamine-conjugated phalloidine). (TIF) [file pone.0029313.s005.tif]

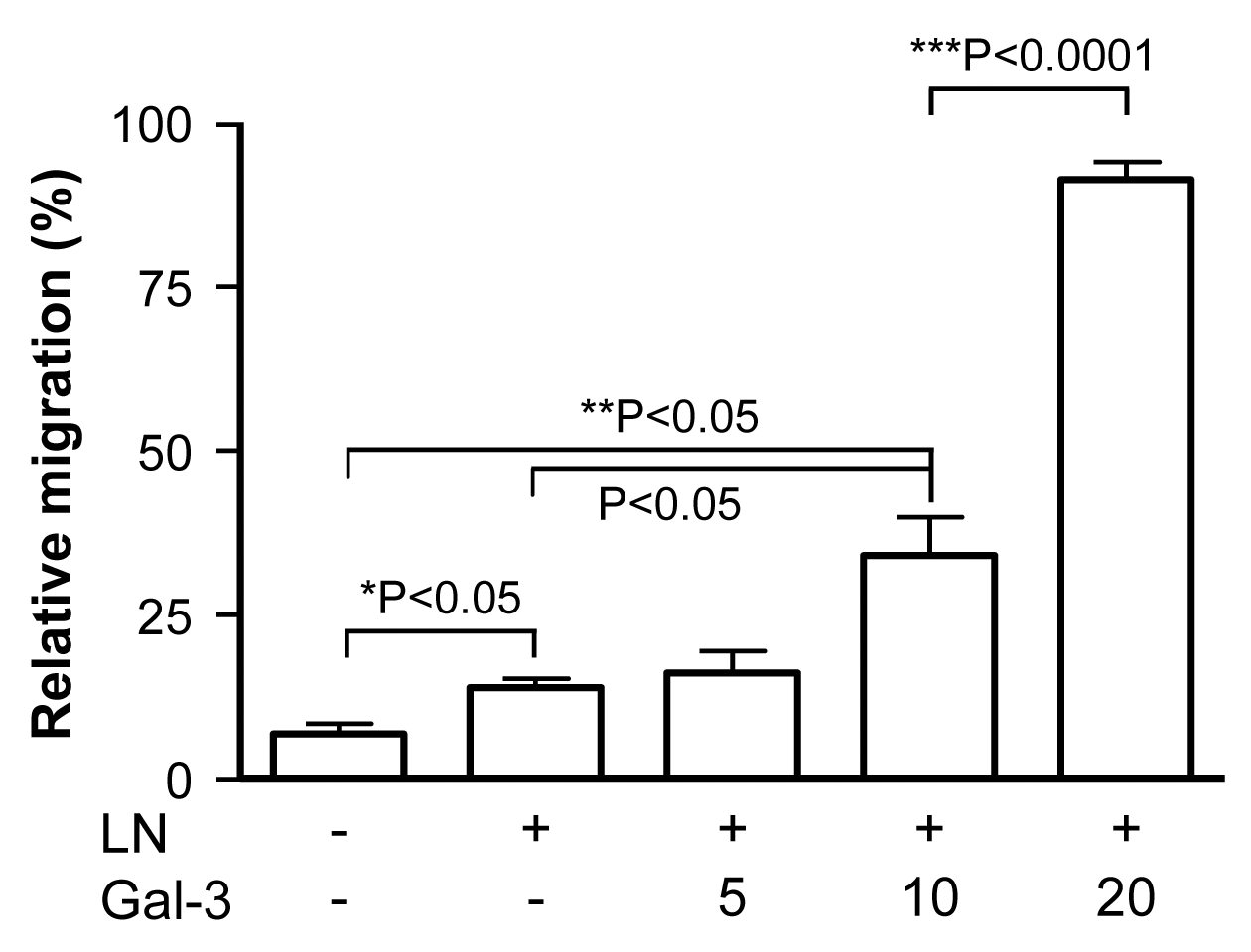

Supplement: Figure S6 — Extracellular galectin-3 increases Σ12 cell migration in a dose-dependent manner. Σ12 cells were grown in coverslips and subjected to the scrape assay. Migration of Σ12 cells into the scratched area was measured after 24 hours by direct counting of DAPI-stained cells using a graticle projected onto micrographs collected using a microscope. Cells were incubated or not in the presence of 10 µg/mL laminin-111 (LN) and 5, 10 or 20 µg/mL galectin-3 (Gal-3), as indicated. Results are means of two independent experiments performed in triplicate with p<0.05 when compared migrating cells in the presence of 5 and 10 µg/mL galectin-3 and p<0.0001 when compared migrating cells in the presence of 20 µg/mL galectin-3 with control cells. (TIF) [file pone.0029313.s006.tif]
